# Preparing for a Bioterrorist Attack: Legal and Administrative Strategies^1^

**DOI:** 10.3201/eid0902.020538

**Published:** 2003-02

**Authors:** Richard E. Hoffman

**Affiliations:** * University of Colorado Health Sciences Center, Denver, Colorado, USA

**Keywords:** bioterrorism, disease outbreaks, public health, jurisprudence, quarantine, patient isolation, perspective

## Abstract

This article proposes and discusses legal and administrative preparations for a bioterrorist attack. To perform the duties expected of public health agencies during a disease outbreak caused by bioterrorism, an agency must have a sufficient number of employees and providers at work and a good communications system between staff in the central offices of the public health agency and those in outlying or neighboring agencies and hospitals. The article proposes strategies for achieving these objectives as well as for removing legal barriers that discourage agencies, institutions, and persons from working together for the overall good of the community. Issues related to disease surveillance and special considerations regarding public health restrictive orders are discussed.

This article proposes and discusses legal and administrative strategies that state and local public health officers and attorneys should consider when preparing for a bioterrorist attack. Through thoughtful preparation, intelligent and enlightened leadership can maximize coordination of available resources in the community.

Two predictable factors will dictate the manner in which state and local governments respond to a bioterrorist attack: 1) the exposure will be covert, and an incubation period will occur before ill persons seek medical care, i.e., there will not be a single location for emergency response by emergency medical teams, law enforcement officers, and firefighters as there was in New York City and Washington, D.C., on September 11, 2001; and 2) the attack will be treated not only as an epidemic but also as an emergency, a crime, and a matter of national security. Because of the second factor, elected political leaders will be in charge of response to the attack, rather than the health commissioner or the state epidemiologist, either of whom would normally manage an epidemic or outbreak control activities. The president, governor, or mayor will assume leadership roles, and public health agencies will need to carry out their duties within an incident command structure.

The magnitude of a bioterrorist attack (i.e., how many persons are exposed to the agent and how many become ill) and the characteristics of the bioagent (e.g., contagious or not) employed by the terrorists are not predictable, but these factors will affect virtually all of the response activities. Nonetheless, “generic” public health duties during a major bioterrorist attack are predictable: providing accurate information to health-care providers and the public about the status of the epidemic and protective measures; conducting disease surveillance and contact tracing; administering vaccines or prophylactic antibiotics; implementing restrictive measures; analyzing human and environmental laboratory specimens; and maintaining the quality of air, water, and food.

To perform these public health duties, two basic objectives must be achieved: 1) a sufficient number of employees and providers in state and local health agencies, hospitals, clinics, and laboratories must show up for work; and 2) a good communications system must exist between staff in the central offices of the public health agency and those in outlying or neighboring agencies and hospitals. Legal and administrative strategies should be developed in advance of an attack with these objectives in mind.

“A sufficient number of employees and providers” includes not only previously trained medical-care providers, laboratory technicians, and public health epidemiologists but also all untrained workers and volunteers who participate in the response to the outbreak. Untrained does not mean unskilled or untrainable. Untrained persons could come from unrelated programs and departments within a public health agency or hospital, from physicians practicing in the community, and from volunteers within and outside the community.

If the number of ill or exposed persons were large, the bioagent were contagious, or both, an epidemic could last for weeks, and the demands on staff could be enormous. For example, in response to a small number of smallpox cases in New York City in 1947, the New York City Department of Health gave smallpox vaccine to over 6 million residents by operating 179 clinics from 9:00 a.m. to 10:00 p.m. 7 days a week for more than 3 weeks and by gaining the support of private physicians, unions, and businesses (*1*). Before October 2001, no one in the United States. had experience with anthrax transmitted through the mail or in the air of postal offices. Many epidemiologic questions arose in the course of responding to that anthrax outbreak, and any future bioterrorist attack will probably result in unpredictable or unimaginable issues despite preparations and training that have been undertaken in the past few years. Thus, the persons responding to an attack will need flexibility in the statutes and regulations that govern disaster emergencies. Rapidly amending statutes and regulations by the usual legislative and administrative processes is not feasible in an emergency; “flexibility” in this sense means the legal authority manifest in statutes and regulations must necessarily be nonspecific and allow for quick action by elected officials and public health leaders. Emergency or executive orders issued by the president, governor, or mayor are the most straightforward legal method for directing response activities customized to the details of a given attack.

In the Colorado bioterrorism statute enacted in 2000, no new powers were authorized for either the health department director or the governor because existing authority to manage emergencies, disasters, and epidemics, though long-standing, was determined to have sufficient latitude to deal with the new threat of bioterrorism. The overall purpose of the introduced bill was to remove legal barriers that discouraged institutions and persons from working together for the overall good of the community. Because the introduced bill did not seek new powers, it was more acceptable to state legislators.

## Obtaining Expert Advice

My experience in state government^1^ has been that after natural or manmade point-source disasters, a governor reflexively turns to the office of emergency management, the department of public safety (or homeland security), or the National Guard for advice and counsel. The staffs of these agencies, however, have neither the expertise necessary to guide the response to an epidemic nor an established, ongoing communications and surveillance system with hospitals, laboratories, and medical providers. It is essential, therefore, to establish a formal process that allows public health and medical experts to assist elected officials in analyzing and interpreting information about the outbreak and in coordinating the public health response to the outbreak. Guidance for fiscal year 2002 supplemental funds for public health preparedness and response for bioterrorism issued by the Centers for Disease Control and Prevention (CDC) requires that a state establish an advisory committee that includes representatives from health departments, first responders, hospitals, and voluntary organizations such as the Red Cross (*11*).

In Colorado this advisory committee includes not only the nine groups listed in the CDC announcement but also the presidents of the state board of health, state medical society, and state hospital association; the state veterinarian; a wildlife disease specialist; a medical examiner; a specialist in posttraumatic stress management; a pharmacist member of the Board of Pharmacy; the Attorney General; the chief public information officer for the state health department; and, as an ex-officio member, the chief of the Colorado National Guard (*3*). These persons were named to the committee because they possess useful expertise or connections to the community. The statute authorizing the formation of the committee provided legal immunity to members for their advice (*4*), and the members pledged that they would attend the committee meetings during a bioterrorist attack rather than report to their regular jobs. By meeting regularly the committee members learn about each other’s skills, experience, and roles and develop a working relationship that, by itself, can be extremely valuable during a crisis.

One notable absence in the composition of the advisory committee is representation from federal agencies, such as CDC, the Federal Emergency Management Agency, the Environmental Protection Agency, and the Federal Bureau of Investigation. Although these agencies cannot, as a practical matter, attend meetings in every state and large municipality, during a crisis they will have an integral role, and disputes are more likely if the leaders are meeting for the first time in a highly stressful situation. For example, local-state-federal disagreements occurred in the management of the pneumonic plague epidemic in Los Angeles in 1924, the last instance of person-to-person transmission of plague in the United States, as well as during the anthrax outbreak in 2001 (*12*,*13*).

## Removing Legal Barriers

Some existing state regulations, which in normal times are intended to ensure quality medical care, could hinder community efforts during a bioterrorist attack. For example, consideration should be given to modifying, for a limited period through executive orders, the regulations that control the prescription and dispensing of medicine, licensing of physicians and nurses, and transfer of patients between hospitals. Providing antibiotics or vaccinations in mass clinics and obtaining the services of retired or out-of-state physicians and nurses may be necessary.

In Colorado, executive orders that address these concerns have been drafted by the governor’s technical advisory committee. The orders would permit a) health-care providers other than pharmacists and physicians, such as nurses and emergency management technicians, to dispense medications, b) medicines to be distributed without an identified patient’s name on the packet or bottle, c) practice of medicine and nursing by professionals who are not currently licensed in Colorado, provided the practice is restricted to caring for epidemic-associated illnesses and the persons are working under the supervision of a licensed practitioner (who is given legal immunity for the supervisee’s work), and d) persons seeking medical care at one facility to be redirected to another facility without initial assessment or stabilization attempt if the initial hospital is unable to care for any more persons or if a specific facility (established or temporary) has been directed to receive epidemic patients, e.g., those with smallpox. These draft orders must still be tailored to the actual emergency and signed by the governor, but the background legal work can be completed ahead of time.

Two additional features of the Colorado bioterrorism statute exist; these features were designed to encourage volunteers and remove legal barriers to cooperation among institutions and agencies. First, the statutory definition of “civil defense worker” was modified to include a “physician, health care provider, public health worker, or emergency medical service provider who is ordered by the governor…to provide specific medical or public health services during and related to an emergency epidemic and who complies with this order without pay or other consideration” (*7*). With this amendment, civil defense workers may receive compensation for injury, including illness caused by bioterrorism, which is suffered as a result of civil defense service. Second, the statute provides that “persons and entities [including hospitals] that in good faith comply completely with board of health rules regarding the emergency epidemic and executive orders…shall be immune from civil or criminal liability for any action taken to comply with the executive order or rule” and that the state shall provide “compensation for property…if the property was commandeered or otherwise used in coping with an emergency epidemic… ” (*4*).

## Requiring Plans for Bioterrorist Events

To ensure that a sufficient number of health-care providers, laboratory technicians, public health epidemiologists, and administrative support workers show up for work during a bioterrorist attack, appropriate personal protection (e.g., respiratory protection, vaccination, or chemoprophylaxis) for the worker and, probably, for household members of the worker are essential. When performing nonstandard work, the worker may also need legal protection, as discussed above. Plans for a bioterrorist attack should include these factors and be written by the employer who knows how the agency operates and is staffed because people work for an agency, hospital, or institution, not a region. Nonetheless, coordination of resources should develop mutual aid agreements with neighboring jurisdictions and integrate single institution or agency plans into community, regional, or statewide plans.

In the 2000 Colorado bioterrorism statute, the state board of health was given the new authority to promulgate rules requiring each state and local health department, general or critical access hospital, and managed-care organization to write a plan for responding to bioterrorism (7). Such rules were adopted in May 2001 (*8*). While hospitals and health departments may have previously written plans for managing mass casualties resulting from aircraft, bus, or train crashes or natural disasters, such plans need to be modified to include consideration of the special circumstances of bioterrorism (e.g., chemoprophylaxis and personal protective equipment for workers, infection control, and handling of laboratory specimens). Because pandemic influenza may pose challenges to the medical and public health systems similar to those of bioterrorism, a single plan for both types of epidemics should be drafted.

## Ensuring Good Communications

During “typical” outbreaks of communicable diseases, clear and timely communication by the state health department with multiple local health departments and hospitals can be a challenge. In a bioterrorist attack, the communications challenge will likely be greater because many more persons and agencies will be involved. The telephone system may not have sufficient capacity for the increased demand or it may be damaged and disorganized, as happened during the response to the attacks on the World Trade Centers in New York City in September 2001 (*14*). Furthermore, a large, sometimes overwhelming, number of inquiries made by members of the public to the public health agency usually occur during public health crises, and therefore, administrative plans for a bioterrorist event should include consideration of this workload.

Legal and administrative strategies should be developed in anticipation of communication challenges. Rather than relying on hospital personnel, public health agencies may find it advantageous to station their own personnel with mobile telephone or radio communications equipment in individual hospitals to assure that public health agencies get the information they need as rapidly as possible. Accomplishing this may require an executive order of the governor that commandeers two-way radios. In Colorado, board of health regulations require the state and local health departments to include assignment of employees to hospitals in the agency’s emergency plan (*8*).

## Disease Reporting and Surveillance

Disease reporting requires specification of what to report in what manner and timeframe to which parties. A first legal step in this process is to require immediate reporting of any suspected or confirmed illness, syndrome, or outbreak caused by any potential bioterrorist agent. For example, Colorado regulations were modified in 1999 so that cases of plague, which had been required to be reported within 24 hours of diagnosis by telephone, fax, or through a Web-based system, were to be reported immediately only by telephone to an on-call person if the physician or hospital suspected the case was related to a bioterrorist event (*9*).

Disease surveillance systems are critical not only for the initial detection of an outbreak but also for monitoring the extent and spread of the outbreak and for determining when it is over. Managing a large outbreak would require gathering information from contact tracing and source-of-exposure investigations as well as information about the availability of critical medicine, medical equipment, and the handling of corpses. These information needs are much different than those needed for early detection of an attack. Therefore, legal authority for surveillance should be modified as necessary to ensure collection of all information that could be needed by the public health agency to fulfill its duties throughout the epidemic. This legal authority may include requirements for groups that do not commonly report information, such as pharmacists, to provide it.

## Restrictive Measures, Isolation, and Quarantine

Administrative public health orders restricting personal behavior of persons with certain diseases, such as tuberculosis, are relatively common in this country (*15*). Such orders are usually hand-delivered to a specific person(s), and the restrictions are removed after a specified period, such as after one incubation period or when an ill person is no longer infectious. Another type of public health order might involve work restriction, e.g., health-care providers who cannot demonstrate evidence of immunity to a vaccine-preventable disease are not permitted to work during an outbreak of such disease.

Few, if any states, however, have experience issuing and enforcing large-scale quarantine orders that last more than 1–2 days. Orders restricting large numbers of contacts of cases of plague to home were issued in Florence, Italy, in 1630 and described in the 1999 book, Galileo’s Daughter (*16*). The enforcement of orders restricting the movement of residents of an entire town in which there was an outbreak of viral hemorrhagic fever was depicted in the 1996 movie, Outbreak. The images of severe disease and enforced quarantine are similar in the book and movie and are plausible and disturbing to lay audiences. A more recent, well-documented example of a large-scale movement restriction was the British epidemic of foot-and-mouth disease of 2001, which affected many farms and businesses and led to the quarantine and slaughter of 4 million sheep, cattle, and pigs for disease control purposes (*17*). In all three examples, a decentralized quarantine was imposed. In general, the advantage of a decentralized strategy (e.g., persons are restricted to home) is that it may reduce the risk for transmission of disease because fewer persons congregate. However, a decentralized strategy may require more community resources to implement and enforce. Alternatively, the centralized strategy (e.g., restricted persons are taken to a sports arena, auditorium, theater, school, or hospital) is seemingly easier for the government to care for restricted persons and to enforce the order but could allow contagious and noncontagious persons to come into contact with each other.

Another example of large-scale quarantine occurred in Los Angeles in 1924 during the last epidemic of pneumonic plague in this country (*12*). Three days after the first 15 cases in this outbreak became known to public health officials, eight city blocks that housed approximately 2,500 Mexicans were placed in quarantine. Public health nurses were sent to the area to make house-to-house inspections to identify new cases, and all patients with suspected cases in the area were examined by physicians at the patient’s home and then sent to the county hospital. The Los Angeles County Board of Charities provided 7-day rations to each household. All persons who lived at addresses where cases had occurred were quarantined in the county general hospital, and a Spanish-speaking priest and social workers were placed in the area to reassure and calm the residents. The quarantine actions taken in this outbreak were a combination of centralized and decentralized strategies.

As has been discussed by Barbera et al. (*18*), numerous concerns regarding large-scale quarantine exist. All states currently have in place varying degrees of legal authority enabling isolation, quarantine, or travel restrictions if needed to maintain the welfare and safety of the public. Drafting restrictive orders in advance is less helpful than with the other types of orders discussed above because restrictive orders require more tailoring to the specific circumstances and parameters of an outbreak. Factors such as duration and location of restriction are dependent on what the bioterrorist agent is, how it is transmitted, how widely the agent has been disseminated, whether exposed persons can be personally identified, and what resources are available to care for restricted persons. Not drafting such orders in advance, however, means that they may be written during the turmoil of multiple agencies trying to control an outbreak. Authorities should never hesitate to revise the orders on the basis of on updated information. At the end of the Operation Topoff exercise, for instance, when the governor had issued a travel restriction order for all of metropolitan Denver and CDC had quarantined the entire state of Colorado, such orders created many unforeseen problems, including how to enforce the orders, maintain essential community services, and distribute foods and prescription medicines. The exercise ended before any of these problems were addressed and resolved.

## Conclusions

Accurate and substantive information given to the public by credible public health and medical experts can do much to allay the fears of the public and encourage their cooperation and participation in constructive, organized community response efforts (*19*,*20*). The foundation for this is thoughtful, detailed preparations. In this article, I have discussed a number of ideas about legal and administrative preparations for a bioterrorist attack, but more work can be done, including development of strategies addressing issues related to mental health, disposal of corpses, performing forensic autopsies, signing death certificates, and managing potential animal vectors of disease.

I have not discussed the sharing of medical and epidemiologic information between public health agencies and law enforcement agencies, such as the Federal Bureau of Investigation. Under normal circumstances, public health officials typically argue that release of disease surveillance information to the criminal justice system will discourage persons with reportable conditions from disclosing to public health officials where they have been and with whom they have had contact. However, a bioterrorist attack is not a routine event, and I recommend that state and local public health agencies review the laws and regulations governing the confidentiality of disease surveillance records and develop a legal and administrative protocol for sharing pertinent and relevant information with law enforcement agencies during a bioterrorist attack (*21*).

Finally, I have not discussed the protection of civil liberties and due process for persons affected by executive orders of the governor and public health officials. This is an important and difficult issue, especially when well persons are quarantined solely on the basis of their having visited, worked, or resided in a particular location at a particular time, as opposed to having had face-to-face contact with a known contagious person. Public health officials and attorneys general should review existing safeguards for the protection of civil liberties and determine whether modifications need to be made for the special circumstances created by a bioterrorist attack.

## Footnotes

1 My perspective is based on experience serving as the state epidemiologist for Colorado from 1987 to 2001; as its chief medical officer from 1998 to 2001; as a participant in Operation Topoff (a full-scale, federal-state-county 4-day bioterrorism exercise conducted in May 2000) (*2*); and as the principal author of Colorado’s bioterrorism response statute (enacted March 2000) (*3*–*8*), its bioterrorism preparedness regulations (adopted May 2001) (*9*), and its bioterrorism reporting regulations (adopted 1999) (*10*).
